# Convergent synthesis of a tetrasaccharide repeating unit of the *O*-specific polysaccharide from the cell wall lipopolysaccharide of *Azospirillum brasilense* strain Sp7

**DOI:** 10.3762/bjoc.10.26

**Published:** 2014-01-29

**Authors:** Pintu Kumar Mandal, Debashis Dhara, Anup Kumar Misra

**Affiliations:** 1 Medicinal and Process Chemistry Division, CSIR-Central Drug Research Institute, BS-10/1, Sector 10, Jankipuram extension, Sitapur Road, Lucknow, 226 031, India; 2 Bose Institute, Division of Molecular Medicine, P-1/12, C.I.T. Scheme VII-M, Kolkata-700054, India

**Keywords:** *Azospirillum brasilense*, glycosylation, lipopolysaccharide, plant growth-promoting bacteria (PGPB), tetrasaccharide

## Abstract

A straightforward convergent synthesis has been carried out for the tetrasaccharide repeating unit of the *O*-specific cell wall lipopolysaccharide of the strain Sp7 of *Azospirillum brasilense*. The target tetrasaccharide has been synthesized from suitably protected monosaccharide intermediates in 42% overall yield in seven steps by using a [2 + 2] block glycosylation approach.

## Introduction

The intensive use of chemicals for the treatment of plant diseases led to environmental pollution, pathogen resistance, an increase in production costs, and serious risks to human health. Among several alternative approaches for the protection of crops against pathogens, biological control based on plant growth-promoting bacteria (PGPB) seems to be among the most promising ones [1]. *Azospirillum brasilense* (*A. brasilense*) is a plant growth-promoting bacterium, found abundant in the rizosphere of several leguminous plants and regular crops such as wheat, rice, corn etc. *Azospirillum* is a Gram-negative rizobacterium, which exerts a variety of beneficial effects to the plant by fixing nitrogen in the soil [2–4]. Besides their nitrogen-fixing capability, *Azospirillum* also produce several vitamins and phytohormones, which promote the overall crop production [5–7]. There are also reports on the moderate biocontrolling capabilities of *Azospirillum* against crown gall disease in plants [8–9], leaf and/or vascular diseases of tomato [10–11] as well as inhibitory properties against the development of bacterial diseases or promoting disease resistance on rice crops [12]. It has been demonstrated that *Azospirilla* produce a diverse range of macromolecules such as exopolysaccharides (EPS), lipopolysaccharides (LPS) and capsular polysaccharides (CPS), which influence their interactions with plants [13–15]. There are only a limited number of reports on structural studies of the LPS present in the cell wall of *Azospirilla* species despite their well-known role in interactions between plants and bacteria [16–21]. Recently, Sigida et al. reported the structure of the repeating unit of the LPS present in the cell wall of *Azospirillum brasilense* strain Sp7: a tetrasaccharide consisting of D-galactose, D-glucosamine, L-rhamnose and L-fucose [22].

**Figure 1:**
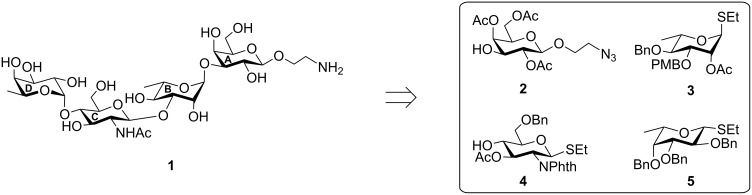
Structure of the synthesized tetrasaccharide and its precursor intermediates. PMB: *p*-methoxybenzyl.

Biological plant growth-promoting agents are becoming attractive as a means for the enhancement of the crop production while minimizing the hazards associated with the use of chemical fertilizers [23–24]. For a detailed understanding of the association of the LPS present in the cell wall of *Azospirillum* in the initial stage of the interactions with plant roots large quantities of the LPS or its repeating unit are required. The dependence on natural sources for a sufficient quantity of the desired tetrasaccharide is inconvenient. Therefore, the development of chemical syntheses facilitate the access to the required tetrasaccharide. A limited number of reports on synthetic studies of the LPS repeating units from the cell wall of *Azospirillum* are available to date [25–29]. A convenient chemical synthesis of the tetrasaccharide repeating unit corresponding to the *O*-specific polysaccharide of the LPS of *A. brasilense* Sp7 strain as its 2-aminoethyl glycoside (Figure 1) is presented herein.

## Results and Discussion

The target tetrasaccharide **1** has been synthesized as its 2-aminoethyl glycoside (Figure 1) to facilitate its conjugation with a suitable substrate (e.g. a protein, a lipid or another aglycone) for biochemical applications. A stereoselective [2 + 2] block glycosylation of the disaccharide derivative **8** with the disaccharide thioglycoside **9** led to the formation of tetrasaccharide **10**, which was finally deprotected to give the desired tetrasaccharide **1**. A number of suitably functionalized monosaccharide intermediates **2**, **3** [30], **4** [31], and **5** [32] were prepared from the reducing sugars by using literature reports. The application of a one-pot reaction sequence for the stereoselective glycosylation and the removal of the *p*-methoxybenzyl (PMB) group [33] as well as the utilization of the “armed–disarmed” glycosylation technique [34–35] are notable points of this synthesis.

**Scheme 1:**
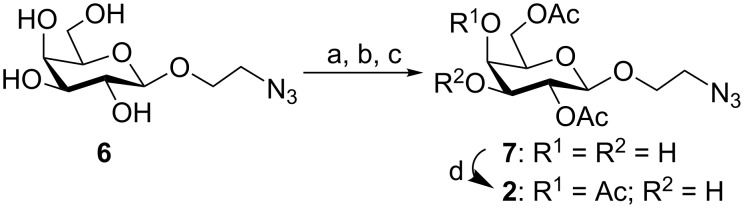
Reagents and conditions: (a) 2,2-dimethoxypropane, *p*-TsOH, DMF, room temperature, 12 h; (b) acetic anhydride, pyridine, room temperature, 6 h; (c) 80% aq AcOH, 80 °C, 1.5 h, 72% overall yield; (d) triethyl orthoacetate, *p*-TsOH, DMF, 2 h then 80% aq AcOH, room temperature, 1 h, 74%.

The transformation of 2-azidoethyl β-D-galactopyranoside (**6**) [36] into 2-azidoethyl 2,6-di-*O*-acetyl-β-D-galactopyranoside (**7**) was carried out in 72% yield by using the reaction sequence consisting of 3,4-*O*-isopropylidenation with 2,2-dimethoxypropane in the presence of *p*-toluenesulfonic acid [37], conventional acetylation of the free hydroxy groups followed by acidic hydrolysis of the acetonide functionality. Selective acetylation of compound **7** by the formation of an orthoester intermediate [38] resulted in the formation of 2-azidoethyl 2,4,6-tri-*O*-acetyl-β-D-galactopyranoside (**2**) in 74% yield (Scheme 1).

**Scheme 2:**
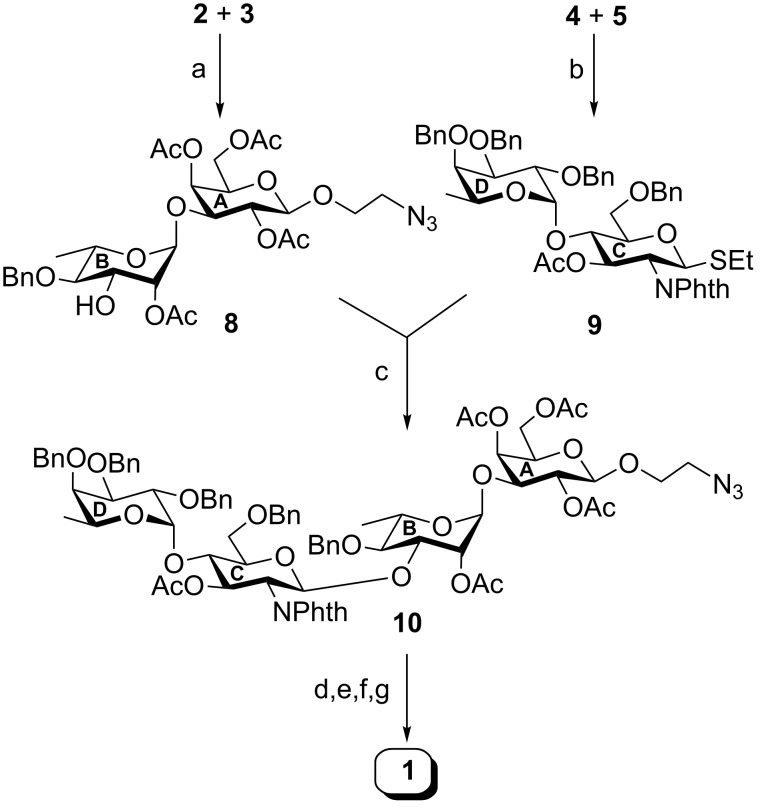
Reagents: (a) *N*-iodosuccinimide (NIS), TfOH, CH_2_Cl_2_, MS 4 Å, −30 °C, 1 h, then 0 °C, 1 h, 77%; (b) NIS, TfOH, CH_2_Cl_2_/Et_2_O 1:1, MS 4 Å, −25 °C, 30 min, 75%; (c) NIS, TfOH, CH_2_Cl_2_, MS 4 Å, −10 °C, 1 h, 72%; (d) NH_2_NH_2_·H_2_O, CH_3_CH_2_OH, 80 °C, 8 h; (e) acetic anhydride, pyridine, room temperature, 1 h; (f) H_2_, 20% Pd(OH)_2_/C, CH_3_OH, room temperature, 24 h; (g) 0.1 M CH_3_ONa, CH_3_OH, room temperature, 3 h, 69% overall yield.

D-Galactosyl acceptor **2** and 3-*O*-PMB-protected L-rhamnosyl donor **3** were coupled with a stereoselective glycosylation promoted by iodonium ions in the presence of a combination of *N*-iodosuccinimide (NIS) and trifluoromethanesulfonic acid (TfOH) [39–40]. The participating 2-*O*-acetyl group in donor **3** ensured the α-selectivity. By raising the temperature after completion of the coupling, the 3-*O*-PMB group was removed in the same pot [33] furnishing disaccharide acceptor **8** in 77% yield. The formation of compound **8** was confirmed by NMR analysis (signals at δ 4.80 (br s, H-1_B_), 4.44 (d, *J* = 8.0 Hz, H-1_A_) and at δ 101.0 (C-1_A_), 99.4 (C-1_B_) in the ^1^H and ^13^C NMR spectra respectively) (Scheme 2).

The 1,2-*cis*-glycosylation of thioglycoside **4** with thioglycoside **5** in the presence of a combination of NIS–TfOH [39–40] in the mixed solvent CH_2_Cl_2_/Et_2_O 1:1 furnished disaccharide thioglycoside derivative **9** in 75% yield. A minor quantity (~8%) of the 1,2-*trans*-glycosylated product was also formed under the reaction conditions but could be removed by column chromatography. The use of Et_2_O in the reaction solvent facilitated the formation of 1,2-*cis* glycoside as the major product. The concept of the Fraser–Reid’s “armed–disarmed” effect was successfully exploited in this glycosylation reaction [34–35]. Compound **5** acted as an activated or armed glycosyl donor because of the presence of an electron-donating benzyl group at the C-2 position. The presence of an electron-withdrawing *N*-phthaloyl group at the C-2 position of compound **4** ensured its functionality as a deactivated or disarmed glycosyl acceptor. The formation of compound **9** was confirmed by its spectroscopic analysis (signals at δ 5.50 (d, *J* = 10.0 Hz, H-1c), 4.97 (d, *J* = 3.5 Hz, H-1_D_), in the ^1^H NMR and δ 100.5 (C-1_D_), 80.6 (C-1_C_) in the ^13^C NMR spectra).

Finally, the 1,2-*trans*-glycosylation of compound **8** with the thioglycoside donor **9** in the presence of NIS–TfOH [39–40] furnished tetrasaccharide derivative **10** in 72% yield. The formation of compound **10** was confirmed by NMR analysis (signals at δ 5.53 (d, *J* = 8.5 Hz, H-1_C_), 4.94 (d, *J* = 3.0 Hz, H-1_D_), 4.85 (br s, H-1_B_), 4.47 (d, *J* = 8.0 Hz, H-1_A_) and at δ 101.1 (C-1_A_), 100.0 (C-1_D_), 98.7 (2 C, C-1_C_, C-1_B_) in the ^1^H and ^13^C NMR spectra respectively). Compound **10** was transformed to the target compound **1** in an overall 69% yield following a sequence of reactions consisting of (a) the conversion of the *N*-phthaloyl group to an acetamido group [41], (b) the hydrogenolysis with hydrogen over Pearlman’s catalyst [42], and (c) the saponification with sodium methoxide. A NMR analysis of compound **1** unambiguously confirmed its formation (signals at δ 4.92 (br s, H-1_B_), 4.85 (d, *J* = 3.5 Hz, H-1_D_), 4.61 (d, *J* = 8.5 Hz, H-1_C_), 4.40 (d, *J* = 7.5 Hz, H-1_A_), and 102.5 (C-1_C_), 102.4 (C-1_A_), 101.9 (C-1_B_), 99.6 (C-1_D_) in the ^1^H and ^13^C NMR spectra, respectively) (Scheme 2).

## Conclusion

In summary, a straightforward and convergent synthesis of the tetrasaccharide **1** as its 2-aminoethyl glycoside corresponding to the *O*-specific polysaccharide of the LPS of *A. brasilense* strain Sp7 has been presented. The use of thioglycosides both as glycosyl donor and acceptor according to the concept of the “armed–disarmed” effect as well as the removal of PMB ether in a one-pot reaction following the glycosylation reduced the overall number of steps.

## Experimental

### General methods

All reactions were monitored by thin-layer chromatography over silica gel-coated TLC plates. TLC spots were visualized by warming plates sprayed with ceric sulfate (2% Ce(SO_4_)_2_ in 5% H_2_SO_4_ in EtOH) on a hot plate. Silica gel 230–400 mesh was used for column chromatography. ^1^H NMR and ^13^C NMR, DEPT 135, 2D COSY, and HSQC spectra were recorded on a Bruker Avance DRX 500 MHz spectrometer by using CDCl_3_ + CCl_4_ and D_2_O as solvents and TMS as an internal reference unless stated otherwise. Chemical shift values δ are expressed in ppm. ESIMS spectra were recorded on a JEOL spectrometer. Elementary analysis was carried out on Carlo ERBA analyzer. IR spectra were recorded on Shimadzu spectrophotometers. Optical rotations were determined on an Autopol III polarimeter. Commercially available grades of organic solvents of adequate purity were used in all reactions.

**2-Azidoethyl 2,6-di-*O*-acetyl-β-D-galactopyranoside (7):** To a solution of compound **6** (3 g, 12.05 mmol) in dry DMF (10 mL) was added 2,2-dimethoxypropane (1.9 mL, 24.09 mmol) followed by *p*-TsOH (150 mg), and the reaction mixture was allowed to stir at room temperature for 12 h. The reaction mixture was neutralized with Et_3_N (1.5 mL) and concentrated under reduced pressure. A solution of the crude product in acetic anhydride and pyridine (10 mL, 1:1; v/v) was kept at room temperature for 6 h, and the solvents were removed under reduced pressure. A solution of the crude product in 80% aq AcOH (100 mL) was allowed to stir at 80 °C for 1.5 h. The solvents were removed under reduced pressure, and the crude product was purified over SiO_2_ by using hexane/EtOAc 4:1 as an eluent to give pure compound **7** (2.9 g, 72%). Colourless oil; [α]_D_^25^ **−**16 (*c* 1.0, CHCl_3_); IR (neat): 3418, 2917, 2857, 1702, 1623, 1585, 1443, 1374, 1316, 1275, 1115, 1069, 1028, 786, 712 cm**^−^**^1^; ^1^H NMR (500 MHz, CDCl_3_) δ 4.96 (dd, *J* = 10.0, 8.0 Hz, 1H, H-2), 4.43 (d, *J* = 8.0 Hz, 1H, H-1), 4.36–4.32 (m, 1H, H-6_a_), 4.27 (dd, *J* = 7.0, 6.5 Hz, 1H, H-3), 4.03–3.99 (m, 1H, H-6_b_), 3.90 (br s, 1H, H-5), 3.68–3.62 (m, 2H, H-4, OC*H*_2_), 3.50–3.45 (m, 2H, OC*H*_2_, C*H*_2_N_3_), 3.27–3.23 (m, 1H, C*H*_2_N_3_), 2.12 (s, 3H, COC*H*_3_), 2.07 (s, 3H, COC*H*_3_); ^13^C NMR (125 MHz, CDCl_3_) δ 171.6 (*C*OCH_3_), 170.9 (*C*OCH_3_), 100.7 (C-1), 72.7 (C-5), 72.5 (C-2), 72.3(C-4), 68.7 (C-3), 68.1 (O*C*H_2_), 62.7 (C-6), 50.6 (*C*H_2_N_3_), 21.0 (CO*C*H_3_), 20.8 (CO*C*H_3_); ESIMS (*m/z*): 356.1 [M + Na]^+^; Anal. calcd for C_12_H_19_N_3_O_8_: C, 43.24; H, 5.75; found: C, 43.13; H, 5.87.

**2-Azidoethyl 2,4,6-tri-*O*-acetyl-β-D-galactopyranoside (2):** To a solution of compound **7** (2.5 g, 7.51 mmol) in dry DMF (10 mL) was added triethyl orthoacetate (5.5 mL, 30.03 mmol) followed by *p*-TsOH (100 mg), and the reaction mixture was allowed to stir at room temperature for 2 h. The reaction mixture was concentrated under reduced pressure, and a solution of the crude product in 80% aq AcOH (80 mL) was stirred at room temperature for 1 h. The solvents were removed under reduced pressure, and the crude product was purified over SiO_2_ by using hexane/EtOAc 4:1 as an eluent to give pure compound **2** (2.1 g, 74%). Yellow oil; [α]_D_^25^ **−**19 (*c* 1.0, CHCl_3_); IR (neat): 3459, 3021, 2927, 2847, 1734, 1555, 1323, 1238, 1133, 1102, 1079, 782, 698 cm**^−^**^1^; ^1^H NMR (500 MHz, CDCl_3_) δ 5.25 (d, *J* = 3.0 Hz, 1H, H-4_A_), 4.92 (dd, *J* = 10.0, 8.0 Hz, 1H, H-2_A_), 4.43 (d, *J* = 8.0 Hz, 1H, H-1_A_), 4.09–4.02 (m, 2H, H-6_abA_), 3.97 (ddd, *J* = 10.6, 4.7, 3.5 Hz, 1H, OC*H*_2_), 3.81–3.74 (m, 2H, H-5_A_, H-3_A_), 3.62 (ddd, *J* = 10.6, 8.4, 3.4 Hz, 1H, OC*H*_2_), 3.43 (ddd, *J* = 13.3, 8.4, 3.5 Hz, 1H, C*H*_2_N_3_), 3.24 (ddd, *J* = 13.3, 4.7, 3.4 Hz, 1H, C*H*_2_N_3_), 2.11 (s, 3H, COC*H*_3_), 2.09 (s, 3H, COC*H*_3_), 1.99 (s, 3H, COC*H*_3_); ^13^C NMR (125 MHz, CDCl_3_) δ 170.9 (*C*OCH_3_), 170.8 (*C*OCH_3_), 170.4 (*C*OCH_3_), 100.8 (C-1_A_), 72.1 (C-5_A_), 71.1 (C-2_A_), 70.9 (C-4_A_), 69.7 (C-3_A_), 68.2 (O*C*H_2_), 61.9 (C-6_A_), 50.5 (*C*H_2_N_3_), 20.9 (CO*C*H_3_), 20.7 (CO*C*H_3_), 20.6 (CO*C*H_3_); ESIMS (*m*/*z*): 398.1 [M + Na]^+^; Anal. calcd for C_14_H_21_N_3_O_9_: C, 44.80; H, 5.64; found: C, 44.62; H, 5.83.

**2-Azidoethyl (2-*O*-acetyl-4-*O*-benzyl-α-L-rhamnopyranosyl)-(1→3)-2,4,6-tri-*O*-acetyl-β-D-galactopyranoside (8):** To a solution of compound **2** (1.5 g, 4.00 mmol) and compound **3** (2.2 g, 4.80 mmol) in anhydrous CH_2_Cl_2_ (10 mL) was added MS 4 Å (2 g), and the reaction mixture was stirred under argon at room temperature for 30 min and cooled to −30 °C. To the cooled mixture were added NIS (1.3 g, 5.76 mmol) and TfOH (30 μL), and the mixture was stirred at the same temperature for 1 h. The temperature of reaction mixture was raised to 0 °C and it was stirred at 0 °C for an additional 1 h. The reaction mixture was filtered through a Celite^®^ bed and washed with CH_2_Cl_2_ (100 mL). The combined organic layer was successively washed with 5% Na_2_S_2_O_3_, satd. NaHCO_3_, and water, dried (Na_2_SO_4_), and concentrated. The crude product was purified over SiO_2_ by using hexane/EtOAc 4:1 as an eluant to give pure compound **8** (2 g, 77%). Yellow oil; [α]_D_^25^ +35 (*c* 1.0, CHCl_3_); IR (neat): 3418, 2911, 2769, 2211, 1664, 1532, 1498, 1411, 1379,1255, 1198, 1063, 982, 661 cm ^−1^; ^1^H NMR (500 MHz, CDCl_3_) δ 7.32–7.25 (m, 5H, Ar-H), 5.28 (d, *J* = 3.0 Hz, 1H, H-4_A_), 5.17 (dd, *J* = 10.0, 8.0 Hz, 1H, H-2_A_), 4.85–4.83 (m, 1H, H-2_B_), 4.80 (br s, 1H, H-1_B_), 4.72 (d, *J* = 11.5 Hz, 1H, PhC*H*_2_), 4.67 (d, *J* = 11.5 Hz, 1H, PhC*H*_2_), 4.44 (d, *J* = 8.0 Hz, 1H, H-1_A_), 4.11–4.05 (m, 2H, H-6_abA_), 4.00–3.96 (m, 1H, OC*H*_2_), 3.86–3.74 (m, 4H, H-5_B_, H-3_B_, H-5_A_, H-3_A_), 3.64–3.61 (m, 1H, OC*H*_2_), 3.49–3.42 (m, 1H, C*H*_2_N_3_), 3.32–3.34 (m, 2H, H-4_B_, C*H*_2_N_3_), 2.11 (s, 3H, COC*H*_3_), 2.10 (s, 3H, COC*H*_3_), 2.09 (s, 3H, COC*H*_3_), 2.03 (s, 3H, COC*H*_3_), 1.27 (d, *J* = 6.2 Hz, 3H, C*C*H_3_); ^13^C NMR (125 MHz, CDCl_3_) δ 170.2 (2 C, *C*OCH_3_), 169.9 (*C*OCH_3_), 169.7 (*C*OCH_3_), 138.3-127.8 (Ar-C), 101.0 (C-1_A_), 99.4 (C-1_B_), 80.9 (C-4_B_), 76.7 (C-3_A_), 74.3 (Ph*C*H_2_), 72.7 (C-3_B_), 71.4 (C-5_A_), 70.3 (C-2_A_), 68.9 (2 C, C-2_B_, C-4_A_), 68.0 (C-5_B_), 67.9 (O*C*H_2_), 61.9 (C-6_A_), 50.5 (*C*H_2_N_3_), 20.9 (CO*C*H_3_), 20.7 (CO*C*H_3_), 20.6 (2C, CO*C*H_3_), 17.9 (C*C*H_3_); ESIMS (*m*/*z*): 676.1 [M + Na]^+^; Anal. calcd for C_29_H_39_N_3_O_14_: C, 53.29; H, 6.01; found: C, 53.10; H, 6.22.

**Ethyl (2,3,4-tri-*O*-benzyl-α-L-fucopyranosyl)-(1→4)-3-*O*-acetyl-6-*O*-benzyl-2-deoxy-2-*N*-phthalimido-1-thio-β-D-glucopyranoside (9):** To a solution of compound **4** (1 g, 2.06 mmol) and compound **5** (1.03, 2.16 mmol) in anhydrous CH_2_Cl_2_/Et_2_O (15 mL, 1:1, v/v) was added MS 4 Å (1 g), and the reaction mixture was allowed to stir at room temperature for 30 min under argon and cooled to −25 °C. To the cooled reaction mixture were added NIS (510 mg, 2.27 mmol) and TfOH (10 μL), and it was stirred at the same temperature for 30 min. The reaction mixture was diluted with CH_2_Cl_2_ (50 mL), filtered through a Celite^®^ bed, and washed with CH_2_Cl_2_. The combined organic layer was successively washed with 5% Na_2_S_2_O_3_, satd. NaHCO_3_, and water, dried (Na_2_SO_4_), and concentrated. The crude product was purified over SiO_2_ by using hexane/EtOAc 5:1 as an eluant to give pure compound **9** (1.4 g, 75%). Yellow oil; [α]_D_^25^ +17 (*c* 1.0, CHCl_3_); IR (neat): 3087, 2856, 1732, 1719, 1625, 1520, 1375, 1239, 1173, 1097, 1072, 989, 911, 823, 755, 698 cm^−1^; ^1^H NMR (500 MHz, CDCl_3_) δ 7.85–7.24 (m, 24H, Ar-H), 5.70 (dd, *J* = 8.5, 1.5 Hz, 1H, H-3_C_), 5.50 (d, *J* = 10.5 Hz, 1H, H-1_C_), 4.97 (d, *J* = 3.5 Hz, 1H, H-1_D_), 4.94 (d, *J* = 12.5 Hz, 1H, PhC*H*_2_), 4.79 (dd, *J* = 12.0 Hz, 2H, PhC*H*_2_), 4.69 (d, *J* = 12.5 Hz, 1H, PhC*H*_2_), 4.63 (dd, *J* = 11.5 Hz, 2H, PhC*H*_2_), 4.39 (dd, *J* = 12.0 Hz, 2H, PhC*H*_2_), 4.27 (t, *J* = 10.0 Hz, 1H, H-2_C_), 4.05 (d, *J* = 11.0 Hz, 1H, H-6_aC_), 3.99–3.97 (m, 1H, H-5_D_), 3.88–3.84 (m, 2H, H-5_C_, H-2_D_), 3.79–3.71 (m, 3H, H-4_C_, H-3_D_, H-6_bC_), 3.60 (br s, 1H, H-4_D_), 2.79–2.57 (m, 2H, SC*H*_2_CH_3_), 1.87 (s, 3H, COC*H*_3_), 1.25 (t, *J* = 7.5 Hz, 3H, SCH_2_C*H*_3_), 1.02 (d, *J* = 6.5 Hz, 3H, CC*H*_3_); ^13^C NMR (125 MHz, CDCl_3_) δ 170.6 (*C*OCH_3_), 167.8, 167.4 (Phth), 138.7–123.5 (Ar-C), 100.5 (C-1_D_), 80.6 (C-1_C_), 79.3 (C-3_D_), 79.0 (C-2_D_), 78.2 (C-4_C_), 77.9 (C-4_D_), 76.7 (C-5_D_), 75.0 (Ph*C*H_2_), 73.9 (2C, C-3_C_, Ph*C*H_2_), 73.2 (Ph*C*H_2_), 72.9 (Ph*C*H_2_), 69.6 (C-6_C_), 67.8 (C-5_C_), 54.2 (C-2_C_), 24.2 (S*C*H_2_CH_3_), 20.9 (CO*C*H_3_), 16.4 (C*C*H_3_), 15.2 (SCH_2_*C*H_3_); ESIMS (*m*/*z*): 924.2 [M + Na]^+^; Anal. calcd for C_52_H_55_NO_11_S: C, 69.24; H, 6.15; found: C, 69.06; H, 6.30.

**2-Azidoethyl (2,3,4-tri-*O*-benzyl-α-L-fucopyranosyl)-(1→4)-(3-*O*-acetyl-6-*O*-benzyl-2-deoxy-2-*N*-phthalimido-β-D-glucopyranoside)-(1→3)-**(**2-*O*-acetyl-4-*O*-benzyl-α-L-rhamnopyranosyl)-(1→3)-2,4,6-tri-*O*-acetyl-β-D-galactopyranoside (10):** To a solution of compound **8** (800 mg, 1.23 mmol) and compound **9** (1.3 g, 1.47 mmol) in anhydrous CH_2_Cl_2_ (10 mL) was added MS 4 Å (500 mg), and the reaction mixture was stirred under argon at room temperature for 30 min and cooled to −10 °C. To the cooled reaction mixture were added NIS (396 mg, 1.76 mmol) and TfOH (6 μL), and it was stirred at same temperature for 1 h. The reaction mixture was filtered through a Celite^®^ bed and washed with CH_2_Cl_2_ (50 mL). The combined organic layer was successively washed with 5% Na_2_S_2_O_3_, satd. NaHCO_3_ and water, dried (Na_2_SO_4_), and concentrated. The crude product was purified over SiO_2_ by using hexane/EtOAc 5:1 as an eluant to give pure compound **10** (1.3 g, 72%). Colorless oil; [α]_D_^25^ +38 (*c* 1.0, CHCl_3_); IR (neat): 3029, 2210, 1849, 1628, 1415, 1322, 1042, 988, 756, 667 cm^−1^; ^1^H NMR (500 MHz, CDCl_3_): δ 7.63–6.86 (m, 29H, Ar-H), 5.59 (dd, *J* = 8.5, 1.5 Hz, 1H, H-3_C_), 5.53 (d, *J* = 8.5 Hz, 1H, H-1_C_), 5.29–5.22 (m, 2H, H-4_A_, H-2_A_), 5.04–5.03 (m, 1H, H-2_B_), 4.94 (d, *J* = 3.0 Hz, 1H, 1_D_), 4.90 (d, *J* = 11.5 Hz, 1H, PhC*H*_2_), 4.85 (br s, 1H, H-1_B_), 4.78 (d, *J* = 12.0 Hz, 1H, PhC*H*_2_), 4.70 (dd, *J* = 12.0 Hz, 2H, PhC*H*_2_), 4.61 (d, *J* = 12.0 Hz, 2H, PhC*H*_2_), 4.57 (d, *J* = 11.5 Hz, 1H, PhC*H*_2_), 4.47 (d, *J* = 8.0 Hz, 1H, H-1_A_), 4.46 (d, *J* = 12.0 Hz, 1H, PhC*H*_2_), 4.35 (dd, *J* = 12.5 Hz, 2H, PhC*H*_2_), 4.24–4.18 (m, 2H, H-2_C_, H-5_A_), 4.17–4.11 (m, 2H, H-6_abA_), 4.10–3.99 (m, 3H, H-4_C_, H-6_aC_, OC*H*_2_), 3.93 (dd, *J* = 10.0, 3.5 Hz, 1H, H-5_D_), 3.87–3.78 (m, 4H, H-2_D_, H-3_A_, H-3_B_, H-5_C_), 3.75–3.69 (m, 4H, H-3_D_, H-5_B_, H-6_bC_, OC*H*_2_), 3.57 (br s, 1H, H-4_D_), 3.55–3.43 (m, 1H, C*H*_2_N_3_), 3.35–3.24 (m, 2H, H-4_B_, C*H*_2_N_3_), 2.29 (s, 3H, COC*H*_3_), 2.18 (s, 3H, COC*H*_3_), 2.05 (s, 3H, COC*H*_3_), 2.00 (s, 3H, COC*H*_3_), 1.79 (s, 3H, COC*H*_3_), 1.03 (d, *J* = 6.0 Hz, 3H, CC*H*_3_), 0.98 (d, *J* = 6.5 Hz, 3H, CC*H*_3_); ^13^C NMR (125 MHz, CDCl_3_) δ 170.5 (*C*OCH_3_), 170.2 (3C, *C*OCH_3_), 169.7 (*C*OCH_3_), 167.7, 167.2 (Phth), 101.1 (C-1_A_), 100.0 (C-1_D_), 98.7 (2C, C-1_C_, C-1_B_), 79.4 (C-3_A_), 79.2 (C-3_D_), 78.2 (C-4_B_), 77.9 (C-4_D_), 76.4 (C-2_D_), 76.1 (C-4_C_), 75.8 (C-5_D_), 75.1 (C-3_B_), 74.9 (Ph*C*H_2_), 73.9 (Ph*C*H_2_), 73.6 (Ph*C*H_2_), 73.3 (C-3_C_), 73.2 (Ph*C*H_2_), 72.9, (Ph*C*H_2_), 71.5 (C-5_A_), 71.4 (C-2_B_), 70.6 (C-2_A_), 68.8 (C-4_A_), 68.4 (O*C*H_2_), 68.2 (C-5_B_), 67.9 (C-6_C_), 67.5 (C-5_C_), 61.9 (C-6_A_), 55.2 (C-2_C_), 50.6 (*C*H_2_N_3_), 21.0 (CO*C*H_3_), 20.9 (CO*C*H_3_), 20.8 (CO*C*H_3_), 20.7 (2 C, CO*C*H_3_), 17.7 (C*C*H_3_), 16.4 (C*C*H_3_); ESIMS (*m*/*z*):1515.2 [M + Na]^+^; Anal. calcd for C_79_H_88_N_4_O_25_: C, 63.53; H, 5.94; found: C, 63.40; H, 6.12.

**2-Aminoethyl (α-L-fucopyranosyl)-(1→4)-(2-acetamido-2-deoxy-β-D-glucopyranoside)-(1→3)-**(**α-L-rhamnopyranosyl)-(1→3)-β-D-galactopyranoside (1):** To a solution of compound **10** (1 g, 0.67 mmol) in EtOH (30 mL) was added hydrazine hydrate (4 mL) and the mixture was allowed to stir at 80 °C for 8 h. The solvents were removed under reduced pressure, and a solution of the crude mass in pyridine (5 mL) and acetic anhydride (5 mL) was kept at room temperature for 1 h. The solvents were evaporated and co-evaporated with toluene under reduced pressure. To a solution of the acetylated product in CH_3_OH (20 mL) was added 20% Pd(OH)_2_/C (200 mg) and the reaction mixture was stirred at room temperature under a positive pressure of H_2_ for 24 h. The reaction mixture was filtered through a Celite^®^ bed, the filtering bed was washed with CH_3_OH (20 mL), and the combined filtrate was concentrated under reduced pressure. A solution of the hydrogenated product in 0.1 M CH_3_ONa in CH_3_OH (10 mL) was allowed to stir at room temperature for 3 h. The reaction mixture was neutralized with Dowex 50W-X8 (H^+^) resin, filtered and concentrated. The crude product was passed through a Sephadex^®^ LH-20 column by using CH_3_OH/H_2_O 3:1 as an eluant to give pure compound **1** (330 mg, 69%). White powder; [α]_D_^25^ +10 (*c* 1.0, H_2_O); IR (KBr): 3456, 2935, 1622, 1356, 1236, 1165, 1067, 697 cm^−1^; ^1^H NMR (500 MHz, D2O) δ 4.92 (br s, 1H, H-1_B_), 4.85 (d, *J* = 3.5 Hz, 1H, H-1_D_), 4.61 (d, *J* = 8.5 Hz, 1H, H-1_C_), 4.40 (d, *J* = 7.5 Hz, 1H, H-1_A_), 4.27–4.24 (m, 1H, H-5_D_), 4.19 (br s, 1H, H-2_B_), 4.04–4.01 (m, 1H, OC*H*_2_), 3.90–3.86 (m, 3H, H-2_D_, H-6_aC_, OC*H*_2_), 3.81–3.78 (m, 2H, H-3_B_, H-6_bC_), 3.77–3.62 (m, 9H, H-2_C_, H-3_C_, H-3_D_, H-4_A_, H-4_D_, H-4_C_, H-5_B_, H-6_abA_), 3.59–3.53 (m, 2H, H-3_A_, H-4_B_), 3.49–3.38 (m, 3H, H-2_A_, H-5_C_, H-5_A_), 3.19–3.16 (m, 2H, C*H*_2_N_3_), 1.93 (s, 3H, COC*H*_3_), 1.16 (d, *J* = 6.5 Hz, 3H, CC*H*_3_), 1.09 (d, *J* = 6.5 Hz, 3H, CC*H*_3_); ^13^C NMR (125 MHz, D2O) δ 175.9 (*C*OCH_3_), 102.5 (C-1_C_), 102.4 (C-1_A_), 101.9 (C-1_B_), 99.6 (C-1_D_), 80.2 (C-3_A_), 79.9 (C-3_B_), 77.2 (C-5_C_), 75.2 (C-5_A_), 75.0 (C-3_C_), 72.4 (C-2_B_), 71.9 (C-4_B_), 70.8 (C-2_A_), 70.2 (C-4_D_), 69.7 (C-3_D_), 69.4 (2C, C-4_A_, C-4_C_), 68.3 (C-2_D_), 68.1 (C-5_B_), 67.0 (C-5_D_), 65.8 (O*C*H_2_), 60.9 (C-6_A_), 59.9 (C-6_C_), 56.2 (C-2_C_), 39.5 (*C*H_2_N_3_), 22.2 (COC*H*_3_), 16.7 (C*C*H_3_), 15.2 (C*C*H_3_); ESIMS (*m/z*): 719.2 [M + H]^+^; Anal. calcd for C_28_H_50_N_2_O_19_: C, 46.79; H, 7.01; found: C, 46.62; H, 7.20.

## Supporting Information

File 1 1D and 2D NMR spectra of compounds **1, 2, 7, 8, 9, 10**.
