# Supplementary material for: Convergent synthesis of a tetrasaccharide repeating unit of the O-specific polysaccharide from the cell wall lipopolysaccharide of Azospirillum brasilense strain Sp7
Source: Beilstein J Org Chem. 2014 Jan 29;10:293–9. doi: 10.3762/bjoc.10.26 (PMC3943683; doi:10.3762/bjoc.10.26)
Supplement: File 1 — 1D and 2D NMR spectra of compounds 1, 2, 7, 8, 9, 10. [file Beilstein_J_Org_Chem-10-293-s001.pdf]

Supporting Information

for

Convergent synthesis of a tetrasaccharide repeating unit of the *O*-specific polysaccharide from the cell wall lipopolysaccharide of *Azospirillum brasilense* strain Sp7

Pintu Kumar Mandal,*[1] Debashis Dhara[2] and Anup Kumar Misra[2]

Address:[1]Medicinal and Process Chemistry Division, CSIR-Central Drug Research Institute, BS-10/1, Sector 10, Jankipuram extension, Sitapur Road, Lucknow, 226 031, India and [2]Bose Institute, Division of Molecular Medicine, P-1/12, C.I.T. Scheme VII-M, Kolkata-700054, India

E-mail: Pintu Kumar Mandal - pintuchem06@gmail.com

*Corresponding author

1D and 2D NMR spectra of compounds **1, 2, 7, 8, 9, 10**

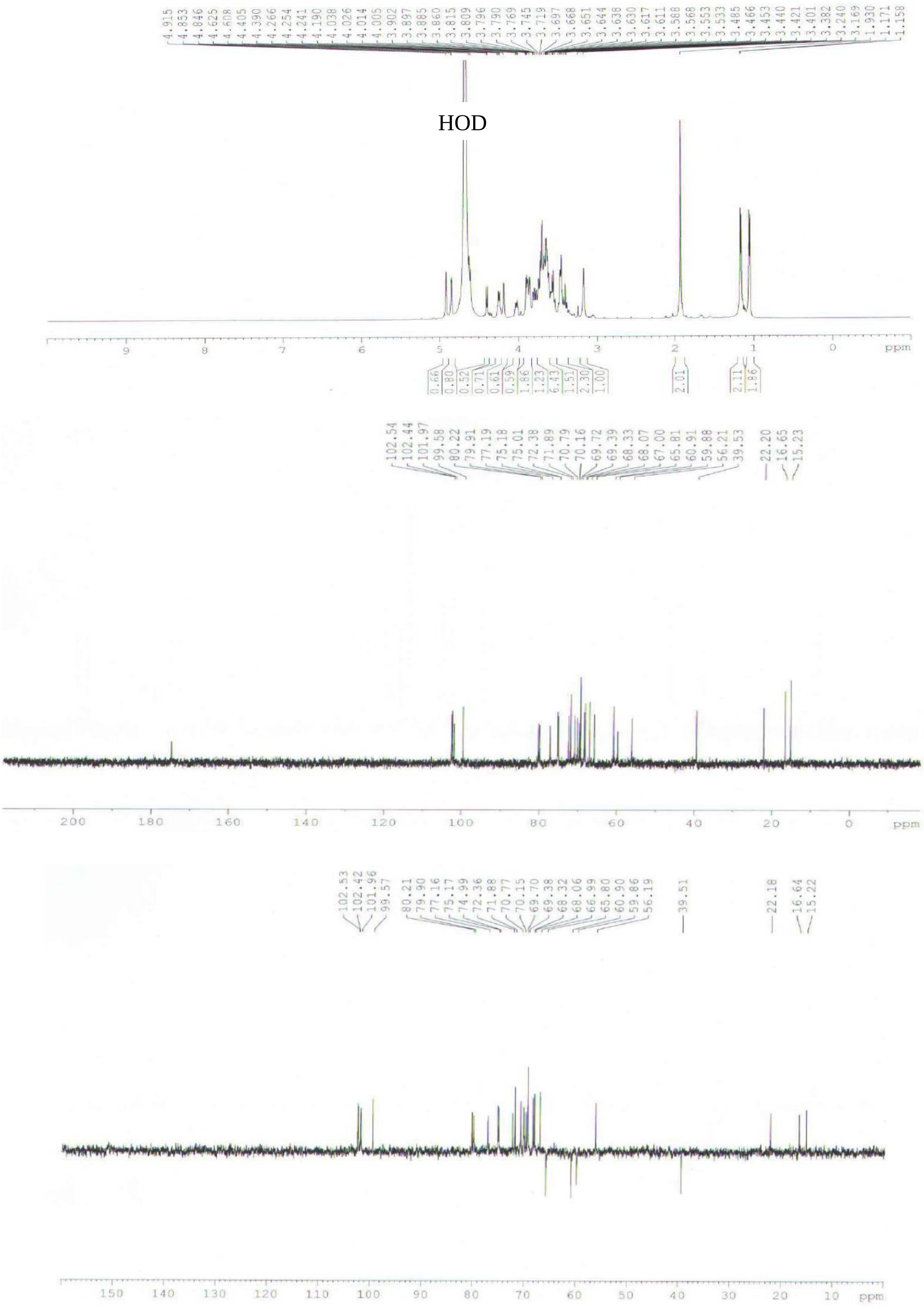

^{1}H, ^{13}C and DEPT 135 NMR spectra of 2-aminoethyl (α-L-fucopyranosyl)-(1→4)-(2-acetamido-2-deoxy-β-D-glucopyranoside)-(1→3)-(α-L-rhamnopyranosyl)-(1→3)-β-D-galactopyranoside **(1)** (D_2O).

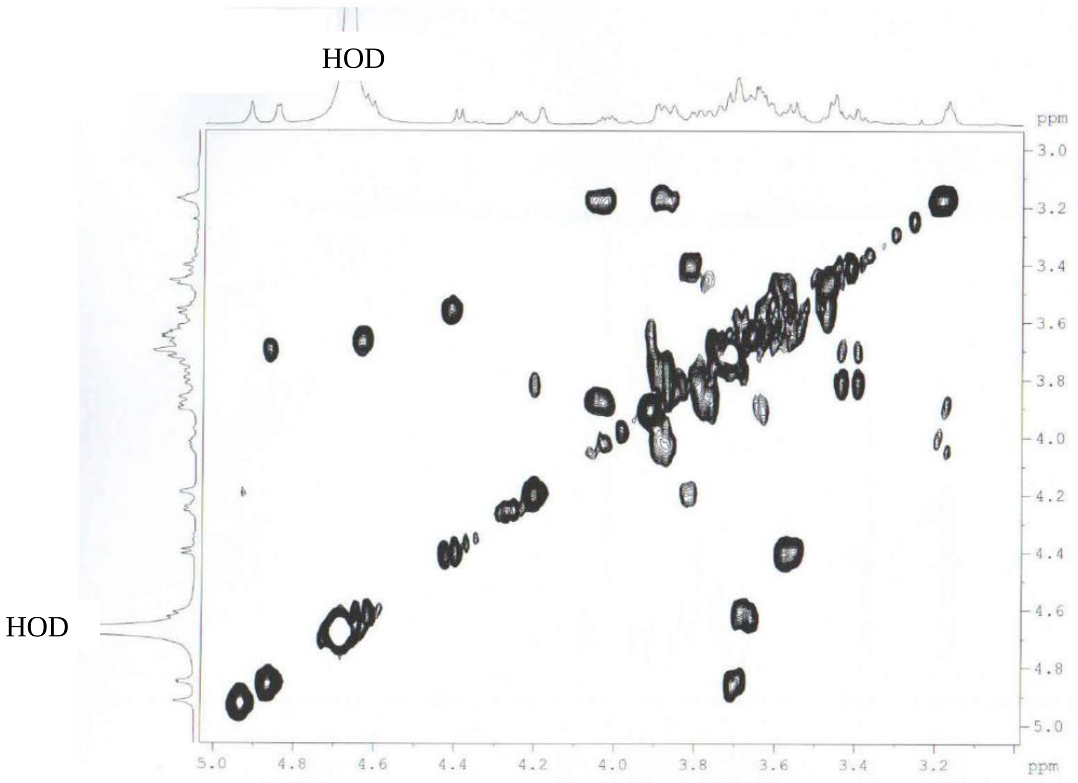

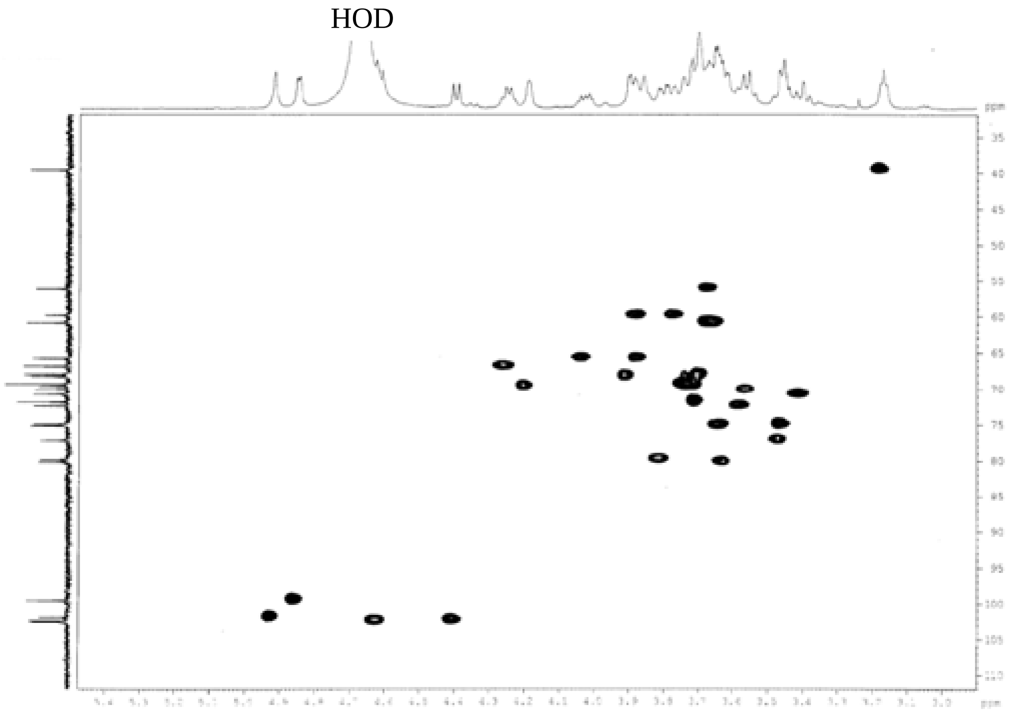

2D COSY and HSQC NMR spectra (selected regions) of 2-aminoethyl (α-L-fucopyranosyl)-(1→4)-(2-acetamido-2-deoxy-β-D-glucopyranoside)-(1→3)-(α-L-rhamnopyranosyl)-(1→3)-β-D-galactopyranoside **(1)** (D_2O).

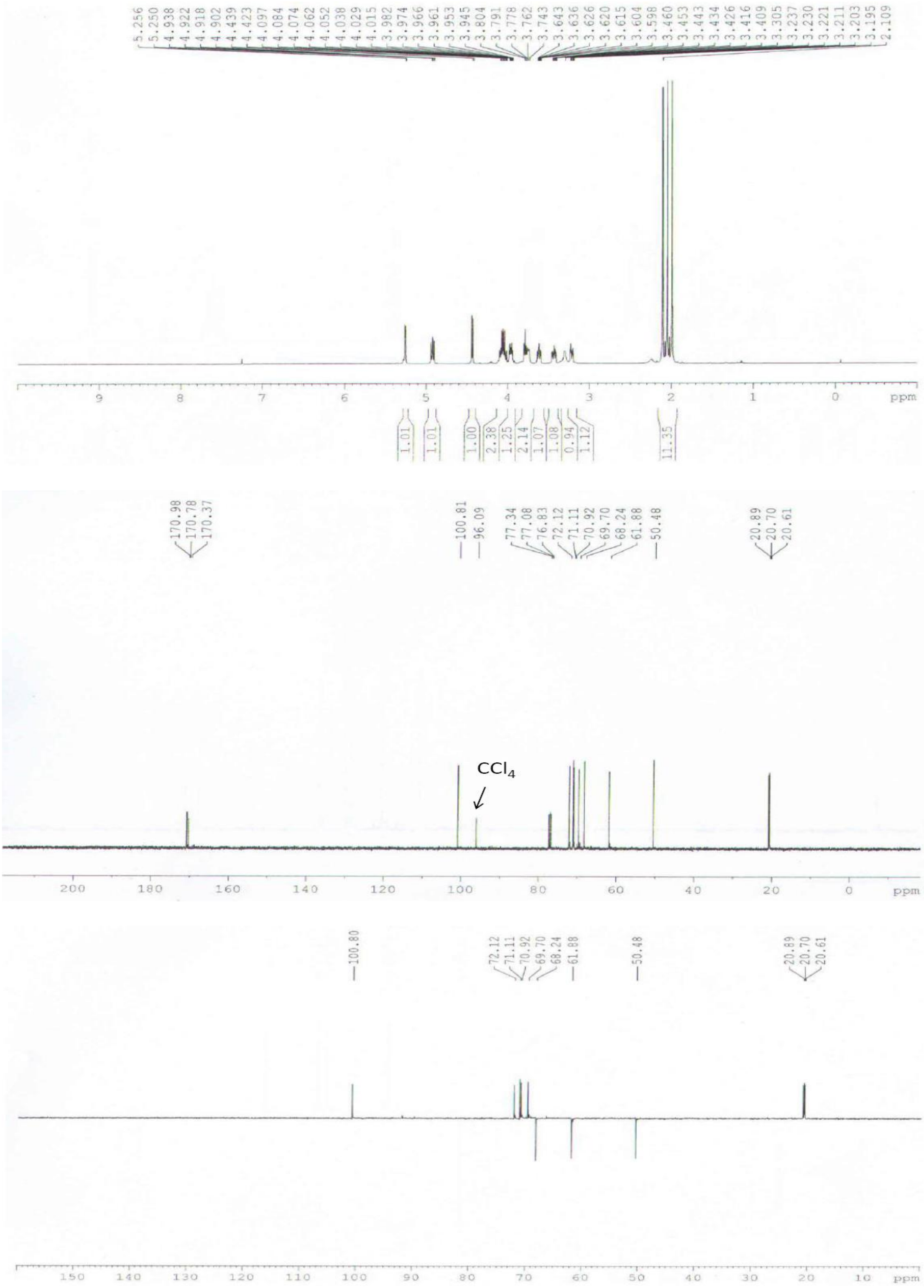

^{1}H, ^{13}C and DEPT 135 NMR spectra of 2-azidoethyl 2,4,6-tri-*O*-acetyl-β-D-galactopyranoside **(2)** ($CDCl_3$ +CCl_4).

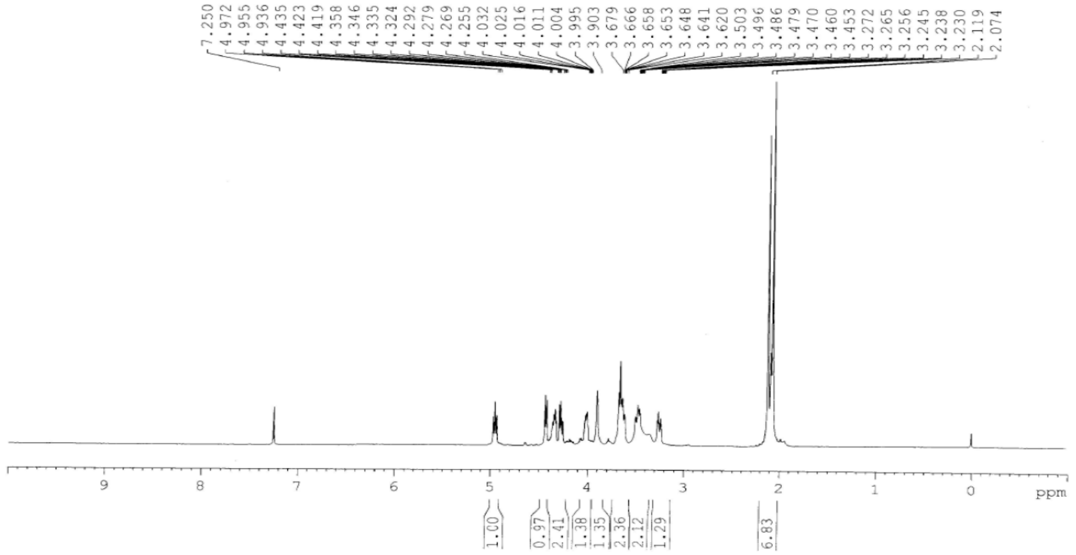

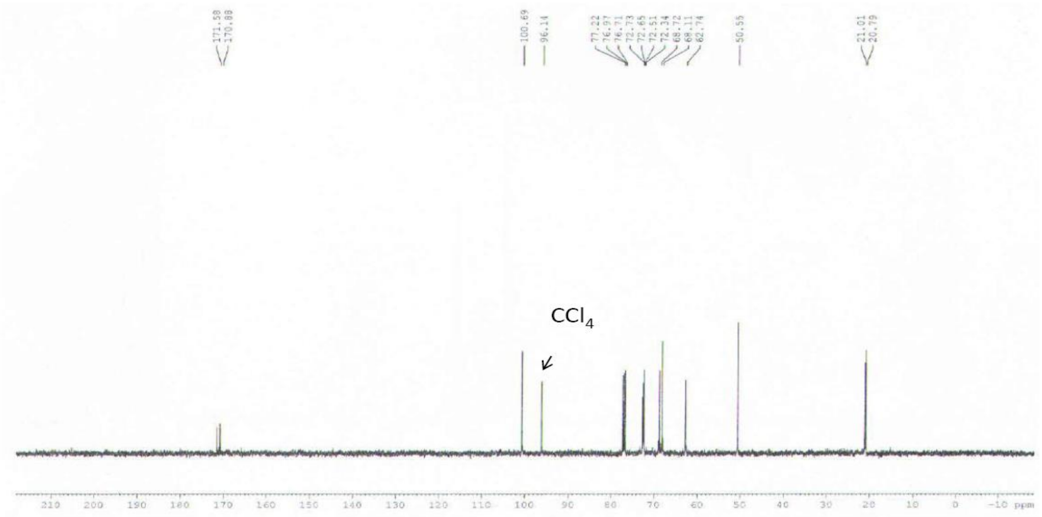

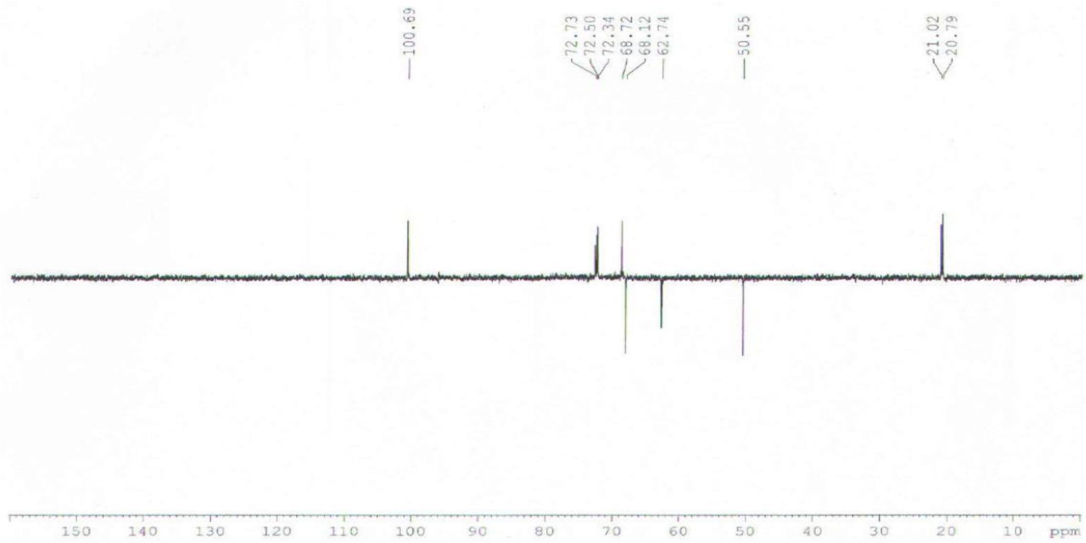

^{1}H, ^{13}C and DEPT 135 NMR spectra of 2-azidoethyl 2,6-di-*O*-acetyl-β-D-galactopyranoside **(7)** ($CDCl_3$ +CCl_4).

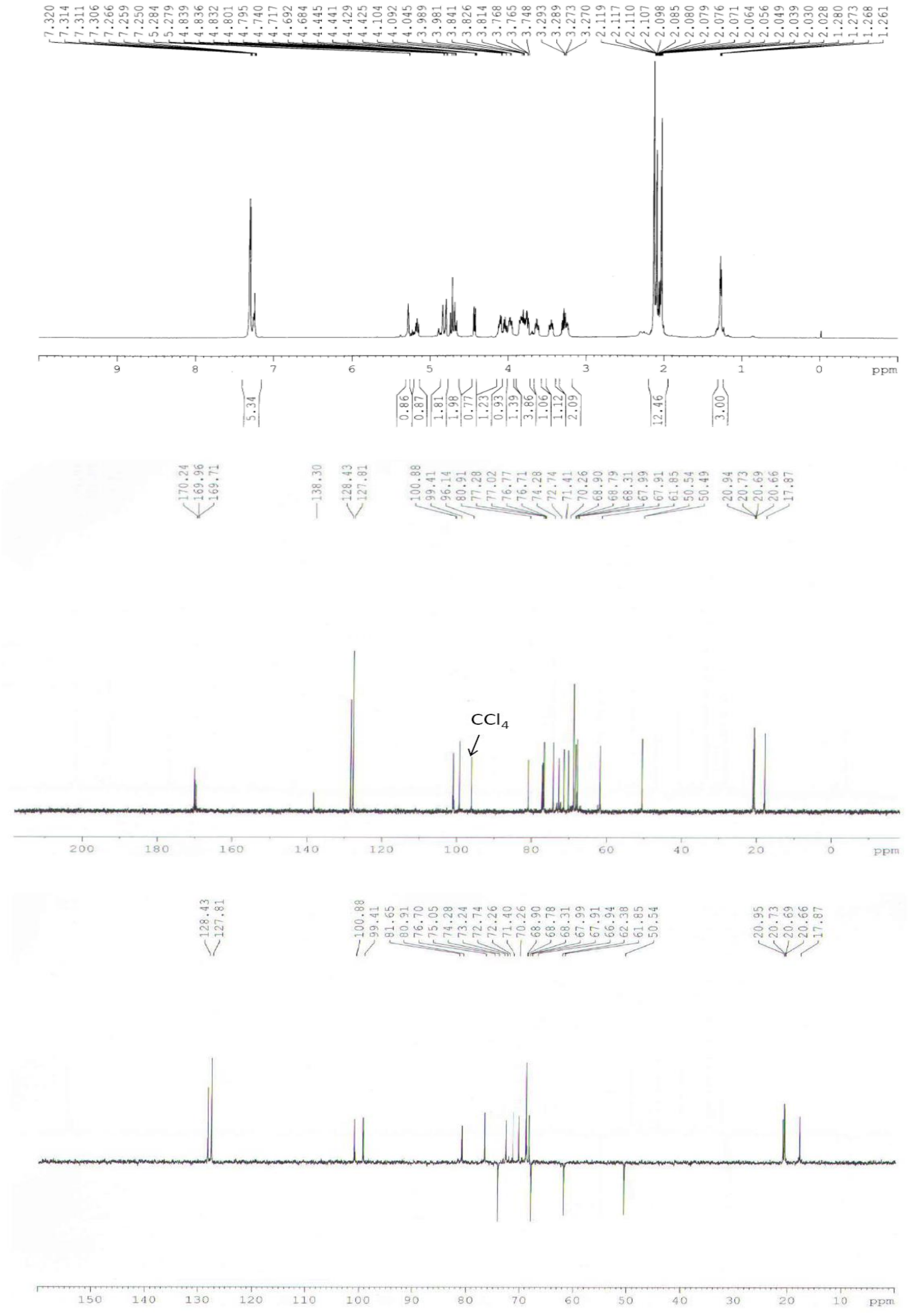

^{1}H, ^{13}C and DEPT 135 NMR spectra of 2-azidoethyl (2-*O*-acetyl-4-*O*-benzyl-α-L-rhamnopyranosyl)-(1→3)-2,4,6-tri-*O*-acetyl-β-D-galactopyranoside (**8**) ($CDCl_3$ +CCl_4).

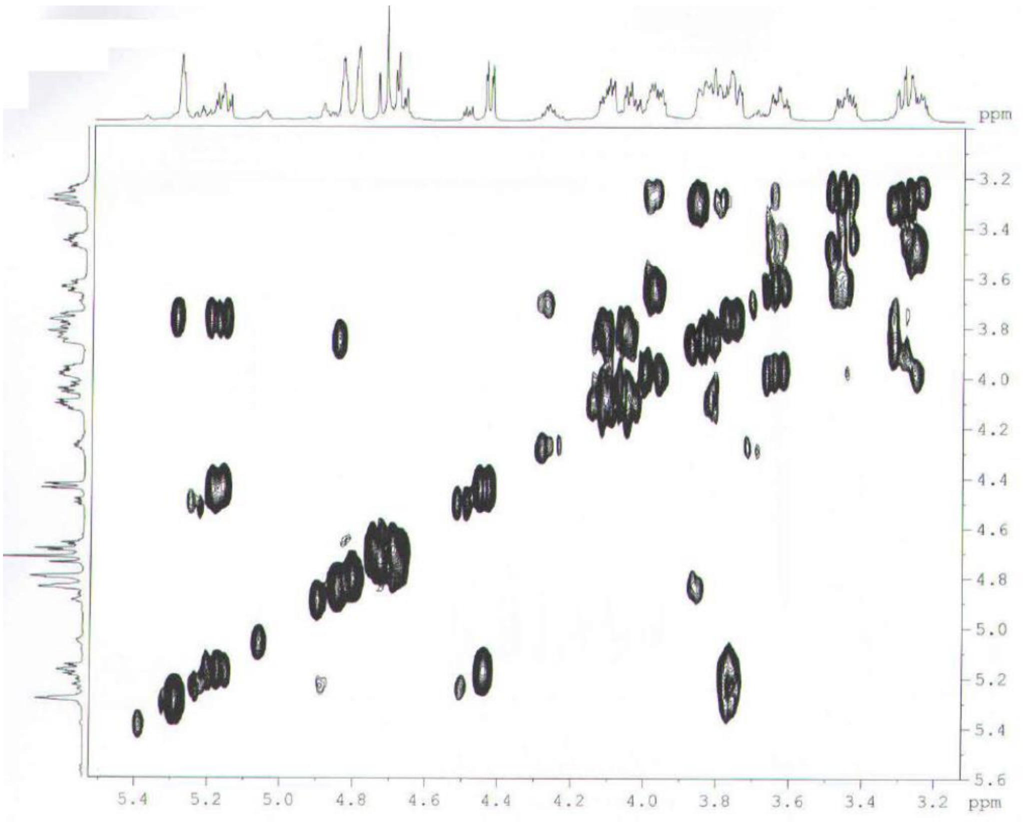

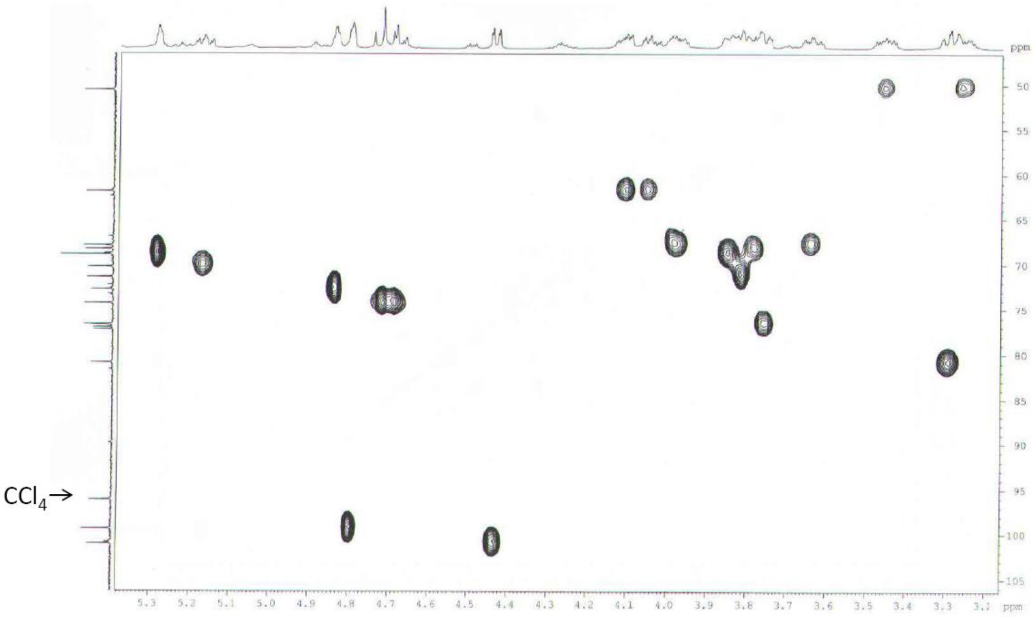

2D COSY and HSQC NMR spectra (selected regions) of 2-azidoethyl (2-*O*-acetyl-4-*O*-benzyl-α-L-rhamnopyranosyl)-(1→3)-2,4,6-tri-*O*-acetyl-β-D-galactopyranoside (**8**) ($CDCl_3$ +CCl_4).

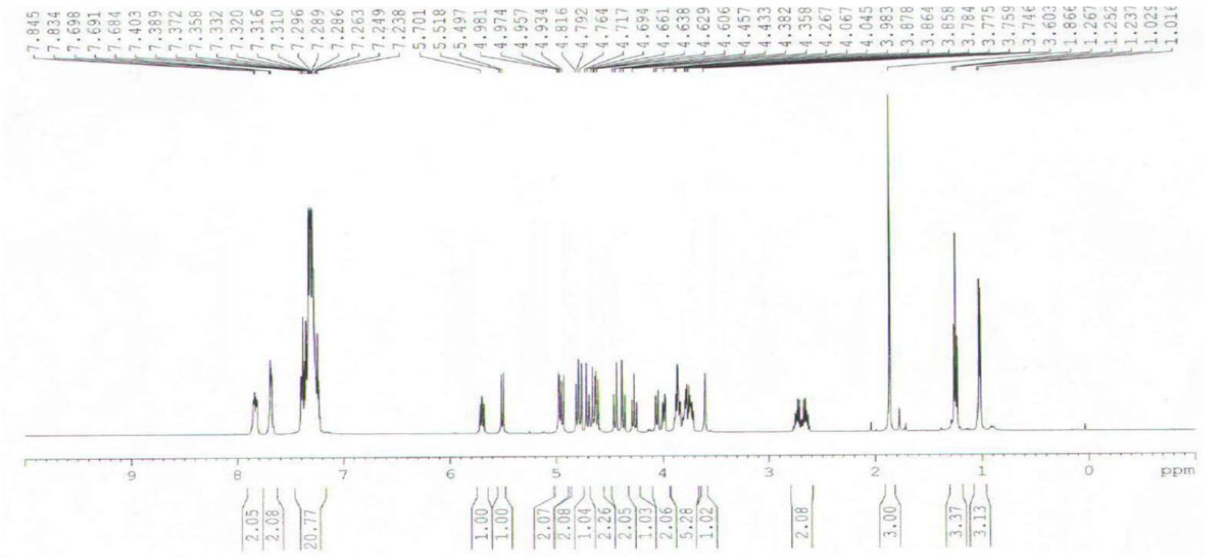

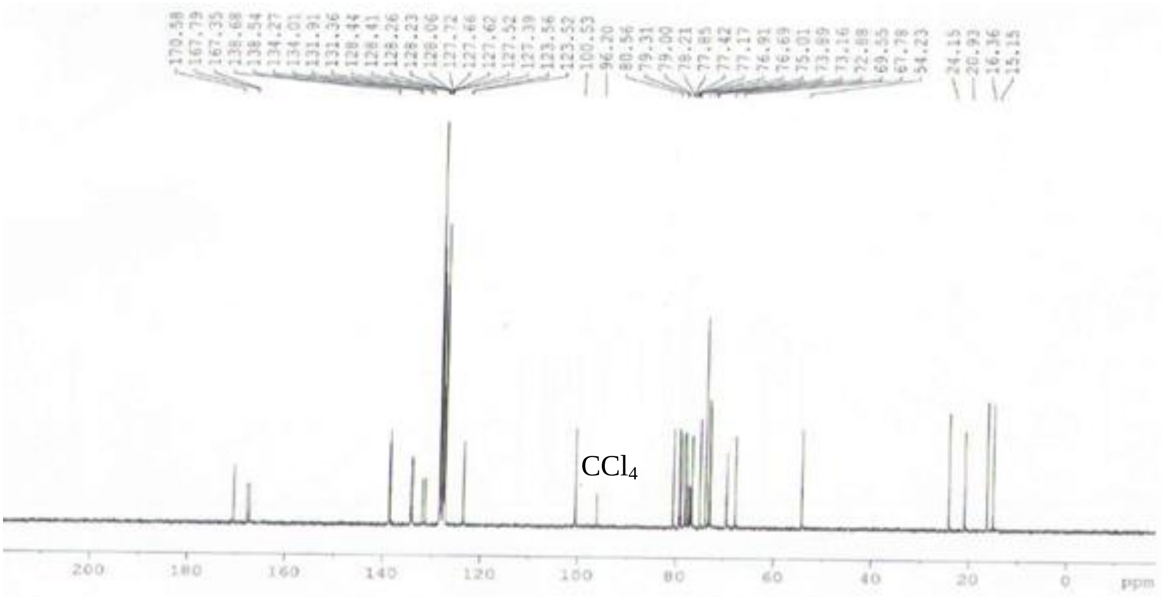

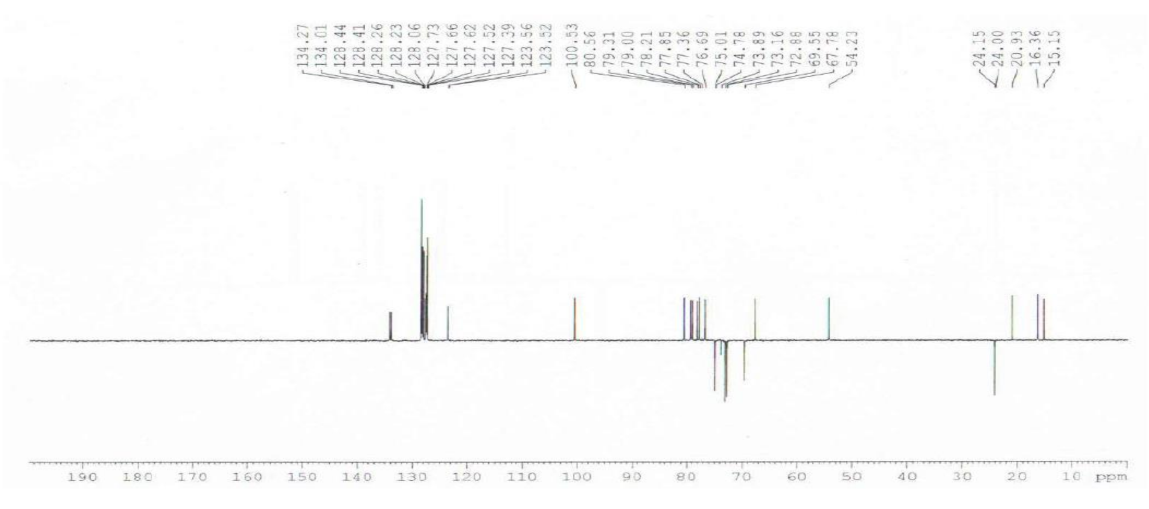

^{1}H, ^{13}C and DEPT 135 NMR spectra of ethyl (2,3,4-tri-*O*-benzyl-α-L-fucopyranosyl)-(1→4)-3-*O*-acetyl-6-*O*-benzyl-2-deoxy-2-phthalimido-1-thio-β-D-glucopyranoside (**9**) ($CDCl_3$ +CCl_4).

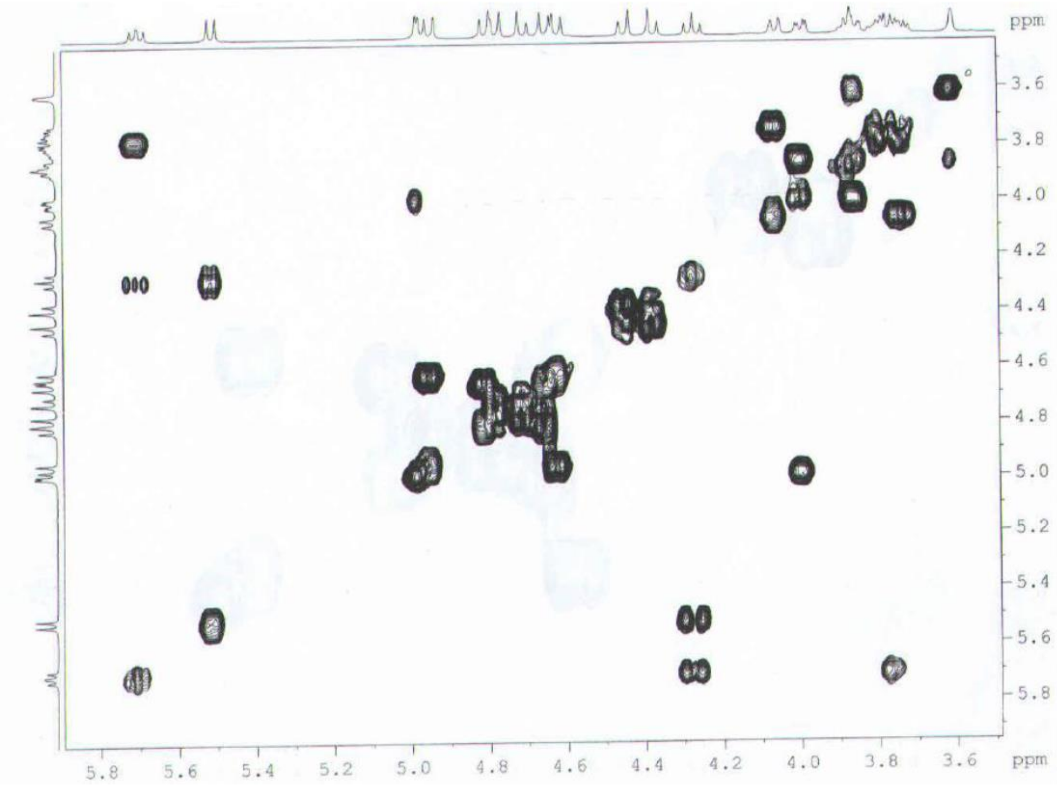

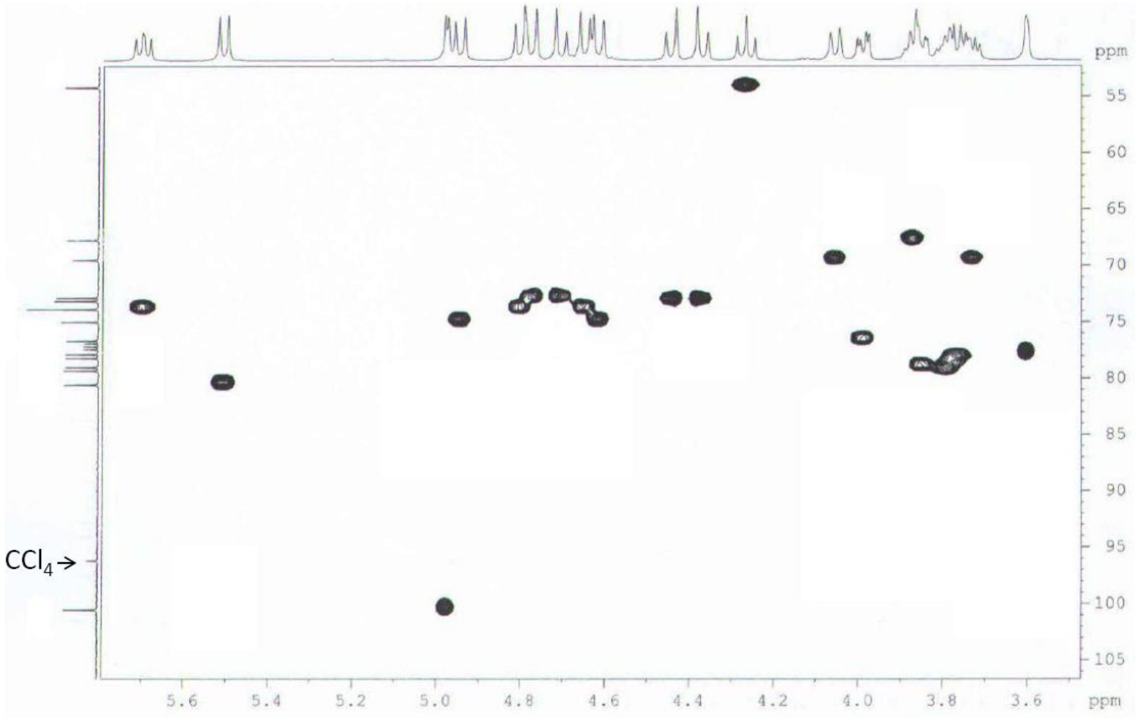

2D COSY and HSQC NMR spectra (selected regions) of ethyl (2,3,4-tri-*O*-benzyl-α-L-fucopyranosyl)-(1→4)-3-*O*-acetyl-6-*O*-benzyl-2-deoxy-2-phthalimido-1-thio-β-D-glucopyranoside (**9**) ($CDCl_3$ +CCl_4).

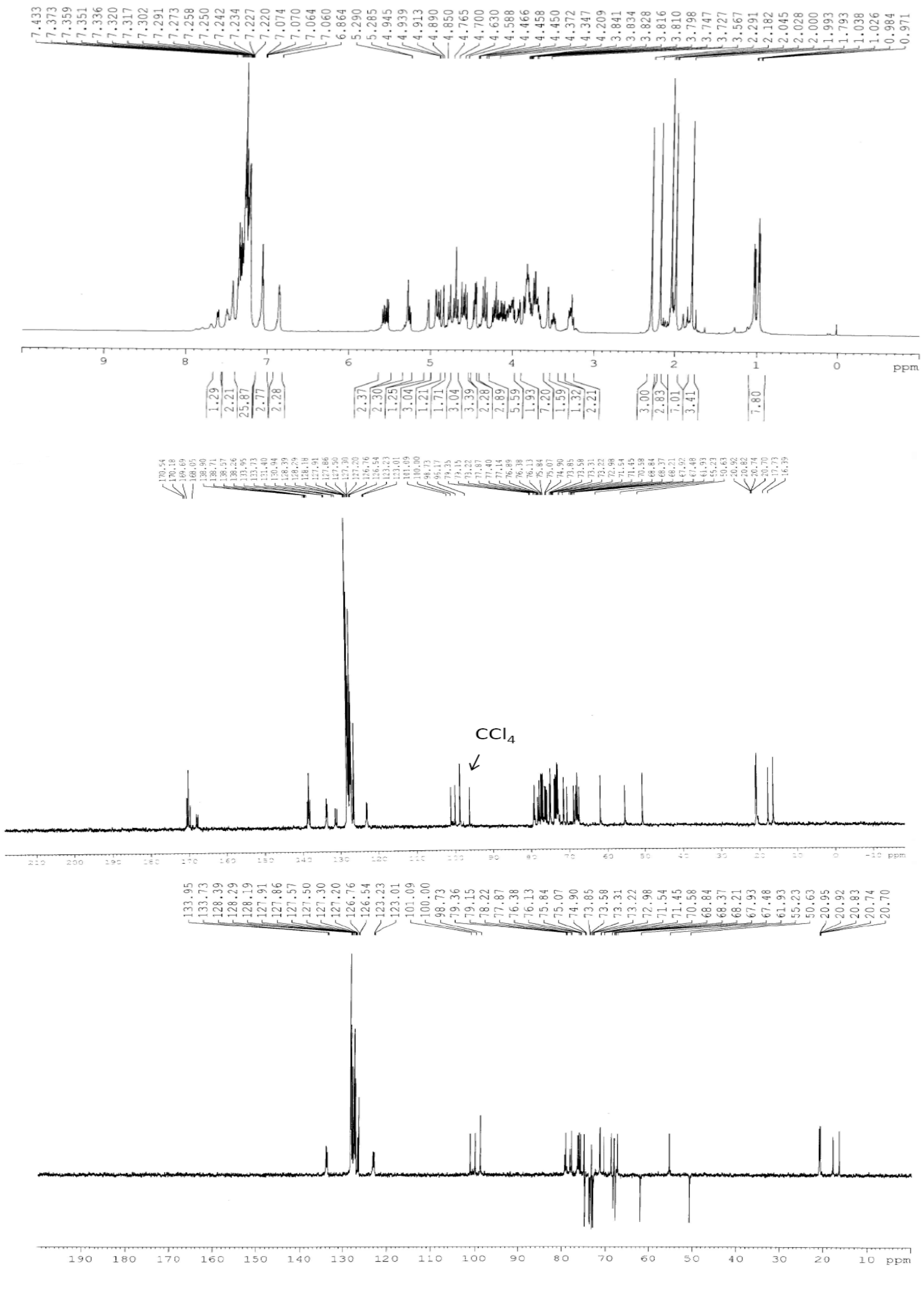

^{1}H, ^{13}C and DEPT 135 NMR spectra of 2-azidoethyl (2,3,4-tri-*O*-benzyl-α-L-fucopyranosyl)-(1→4)-(3-*O*-acetyl-6-*O*-benzyl-2-deoxy-2-phthalimido-β-D-glucopyranoside)-(1→3)-(2-*O*-acetyl-4-*O*-benzyl-α-L-rhamnopyranosyl)-(1→3)-2,4,6-tri-*O*-acetyl-β-D-galactopyranoside (**10**) ($CDCl_3$ +CCl_4).

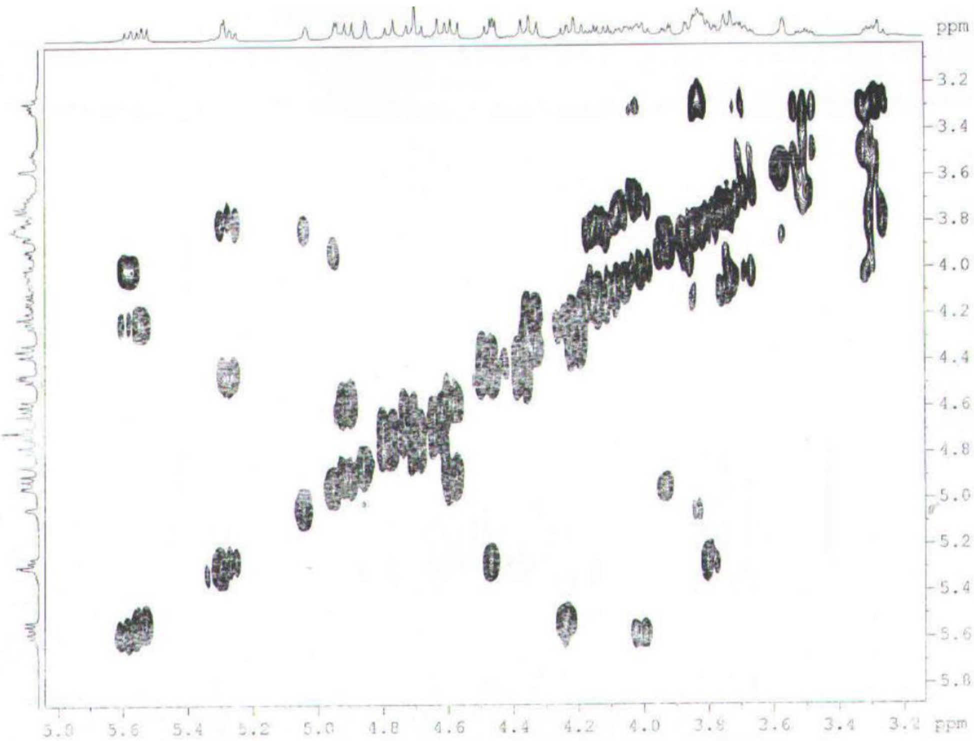

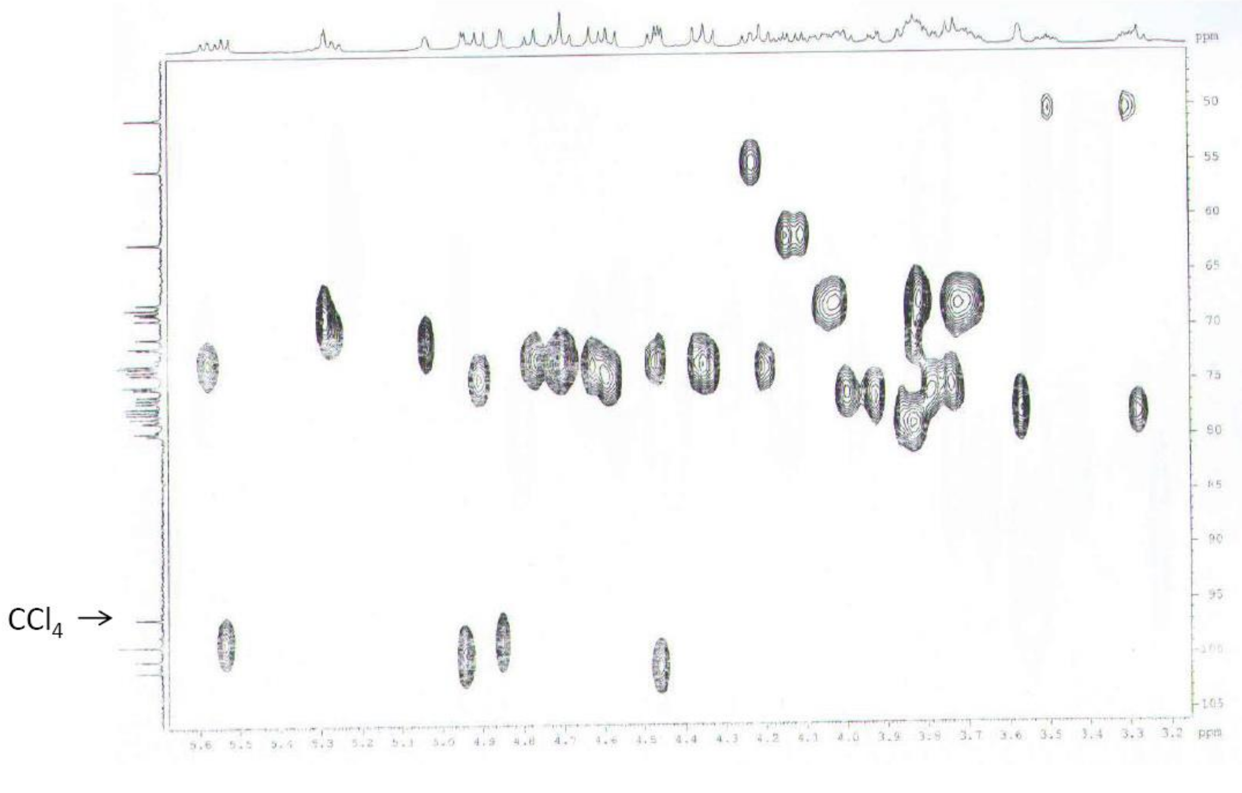

2D COSY and HSQC NMR spectra (selected regions) of 2-azidoethyl (2,3,4-tri-*O*-benzyl-α-L-fucopyranosyl)-(1→4)-(3-*O*-acetyl-6-*O*-benzyl-2-deoxy-2-phthalimido-β-D-glucopyranoside)-(1→3)-(2-*O*-acetyl-4-*O*-benzyl-α-L-rhamnopyranosyl)-(1→3)-2,4,6-tri-*O*-acetyl-β-D-galactopyranoside (**10**) ($CDCl_3$ +CCl_4).
